# Modeling Development in Retinal Afferents: Retinotopy, Segregation, and EphrinA/EphA Mutants

**DOI:** 10.1371/journal.pone.0104670

**Published:** 2014-08-14

**Authors:** Keith B. Godfrey, Nicholas V. Swindale

**Affiliations:** 1 NERF, Leuven, Belgium; 2 imec, Leuven, Belgium; 3 Canadian Centre for Behavioral Neuroscience, University of Lethbridge, Alberta, Canada; 4 Department of Applied Mathematics and Theoretical Physics, University of Cambridge, Cambridge, United Kingdom; 5 Department of Ophthalmology and Visual Sciences, University of British Columbia, Vancouver, Canada; Tokai University, Japan

## Abstract

During neural development, neurons extend axons to target areas of the brain. Through processes of growth, branching and retraction these axons establish stereotypic patterns of connectivity. In the visual system, these patterns include retinotopic organization and the segregation of individual axons onto different subsets of target neurons based on the eye of origin (ocular dominance) or receptive field type (ON or OFF). Characteristic disruptions to these patterns occur when neural activity or guidance molecule expression is perturbed. In this paper we present a model that explains how these developmental patterns might emerge as a result of the coordinated growth and retraction of individual axons and synapses responding to position-specific markers, trophic factors and spontaneous neural activity. This model derives from one presented earlier (Godfrey et al., 2009) but which is here extended to account for a wider range of phenomena than previously described. These include ocular dominance and ON-OFF segregation and the results of altered ephrinA and EphA guidance molecule expression. The model takes into account molecular guidance factors, realistic patterns of spontaneous retinal wave activity, trophic molecules, homeostatic mechanisms, axon branching and retraction rules and intra-axonal signaling mechanisms that contribute to the survival of nearby synapses on an axon. We show that, collectively, these mechanisms can account for a wider range of phenomena than previous models of retino-tectal development.

## Introduction

The development of neural connections is characterized by immature neurons extending axons to their target areas in the brain. During development these axons extend, branch and retract and their synapses onto target dendrites also form and retract. Despite this complex behavior observed in individual axons, collectively these axons generate stereotypical arbors and establish regular patterns of connectivity onto target cells that are observable at the network level. In the visual system, these patterns include retinotopic organization and the segregation of afferents based both on eye of origin and the firing properties of ON and OFF retinal ganglion cell (RGC) types [1]–[5]. In studies where guidance molecule expression is altered, characteristic perturbations of retinotopic organization are also observed (e.g., [6]–[10]). These organizational patterns occur in both retinocollicular and retinogeniculate pathways [2], [5]–[9], [11]–[14].

Neural connectivity is defined by synapses and synapse presence is physically constrained by the location of axons. An understanding about how factors such as neural activity and molecular guidance mechanisms can generate these stereotypic patterns of connectivity thus requires understanding how these mechanisms guide individual axons and synapses to collectively generate observed patterns of organization. Synapse and axon development are influenced by many underlying phenomena. Axon growth is influenced by the presence of synapses [15] and trophic factors affect both axon and synapse growth and stability [16]–[20]. Guidance molecules expressed on retinal neurons and their targets influence axon growth [21]–[23] and synapse stability and plasticity [24]–[27]. Patterned spontaneous activity [28]–[30] helps to guide synapse segregation and axon refinement [31]–[34] and homeostatic mechanisms regulate the strength of synaptic connectivity [35]. Few models have so far attempted to encompass this range of factors. This study presents a computational model that shows how these cellular behaviors can account for retinotopic organization, ocular dominance and ON-OFF segregation.

The computational model described here is based on the general description, structure and assumptions as a previous computational modeling study (GES-2009, [36]). The previous model showed how the above cellular behaviors could govern synapse and axon growth and could account for the development of retinotopic maps. The present study goes beyond the previous work in several ways. First, the model described here represents the same cellular behaviors as GES-2009 but in a mathematically and mechanistically distinct way (e.g., axons used a resource-based growth algorithm in GES-2009 and a probabilistic algorithm here – see Methods) while preserving their qualitative description and behavior. Both models demonstrate similar patterns of retinotopic map development, suggesting that the described patterns of retinotopic organization and axon arbor refinement are emergent properties of the above phenomena. The similar behavior of the models suggests that it is the qualitative aspects of these represented behaviors that is important for retinotopic development and not a specific mechanistic or mathematical implementation. Second, the present study greatly expands upon the data these cellular behaviors are able to account for, including developmental patterns observed after the up- and down-regulation of guidance molecules as well as the activity-dependent segregation of retinal afferents. Segregation appears to occur as a result of temporally distinct patterns of activity between afferents, whether this is from different retinas or from the temporally offset firing properties of ON and OFF RGCs [37]. Developmental perturbations that are observed when molecular guidance expression is altered (e.g., [6]–[10], [38]) are explained by increased and decreased chemospecificity when guidance molecule expression is up- and down-regulated, respectively.

The results from the computational model in this study were compared to results obtained from GES-2009, a related model that represented the same general description and explicit assumptions. While both models shared many behaviors they also exhibited subtle differences and generated different predictions. These differences are an expected outcome when a computational model represents a descriptive biological hypothesis. The process of converting a biological description to mathematical form introduces implicit (hidden) assumptions in the mathematical model, and those assumptions affect model output. The results of this study suggest that it may be useful to compare different models representing the same hypothesis when generating experimentally testable predictions, and that mathematical formula variation, as well as parameter variations, should be explored to demonstrate the robustness of a complex model.

## Results

The model presented here is based on experimental observations made in a variety of structures and species, including Xenopus tectum, mouse colliculus, mammalian LGN and cerebral cortex. Hence, the model is generic in the sense that it describes phenomena not necessarily all found in the same retinal target structure. For simplicity of description, the model is most closely based on the paradigm of retinocollicular (retinotectal) development as is observed in mouse and chick [23] with RGC axons initially overshooting their target area and, through a process of axon branching and refinement, later establishing refined connections in the retinotopically correct area of the colliculus. Developmental phenomena observed elsewhere in the visual system, such as eye-specific segregation as observed in the lateral geniculate nucleus (LGN) and cortex, and the segregation of ON and OFF RGCs that are observed in the LGN, are examined under this paradigm. Axon development in the LGN follows a similar developmental pattern to that observed in mouse colliculus, with initial target overshoot and subsequent branching, and this developmental pattern also occurs in many areas of the cortex [39], [40]. The model has intentionally been designed so that it is capable of adaptation to modeling structures (such as mammalian visual cortex) where ocular dominance columns and ON-OFF segregation occur. The model is not species-specific.

**Figure 1:**
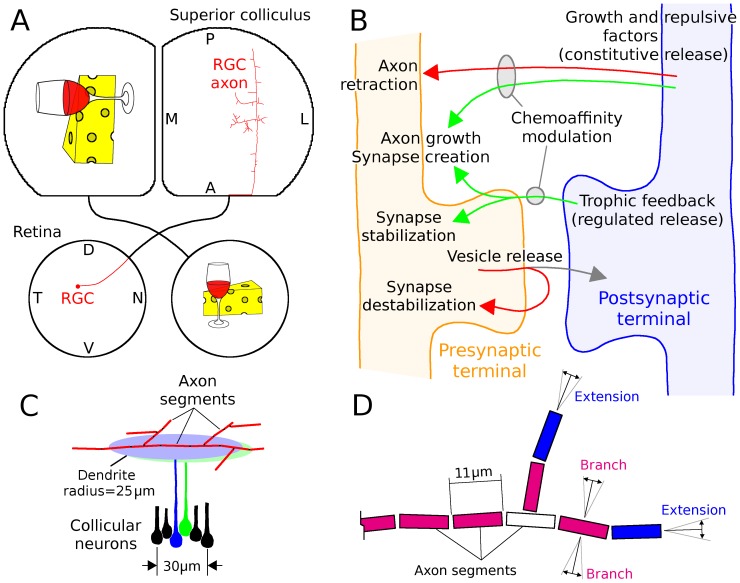
Model structure. *A*, Cartoon of simulated retinocollicular projection. Each retina extends axons to the contralateral colliculus. The model simulated the development of individual RGC axons from when they first grew through the colliculus until a refined arbor was produced. Each axon was assumed to have a retinotopically correct target in the colliculus that was indicated by guidance molecules. *B*, Conceptual organization of the model. Constitutive dendritic release of growth and repulsive factors (e.g., neurotrophins and pro-neurotrophins) influenced the growth and retraction of axons and the local growth of synapses on these axons. Regulated postsynaptic release of trophic factors stabilized synapses and enhanced axon and synapse growth on the presynaptic axon near point of receipt. Vesicle release destabilized synapses, resulting in their eventual retraction if they did not receive sufficient trophic feedback. Trophic factor was provided when a postsynaptic spike followed within tens of milliseconds of vesicle release. The chemoaffinity of an axon segment to its surroundings, regulated by ephrin and Eph receptors and ligands in vivo [23], modulated the efficacy of growth and repulsive factors on each axon based on the co-localization of these molecules [55]–[59]. This meant that growth and trophic factors had higher efficacy on an axon in the retinotopically correct area of the colliculus compared to one further removed, and vice versa for repulsive factors. *C*, Simulated RGC axons in the colliculus were composed of segments 11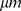 long that could each extend, branch and retract. Collicular neurons were densely packed (167/mm, 27,900/mm^2^) and each had a dendritic field 50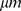 in diameter. Development was represented in two dimensions and each dendritic field was treated as a disk. Axon segments could generate synapses with any dendritic field that it overlapped with. *D*, Cartoon of axon, showing axon segments, extension and branching. Axon extension occurred at axon tips (i.e., segments that had no children, in blue) and branching occurred in segments that had already extended but that did not have any branches (red). Axon retraction occurred only at axon tips. Extending axons grew in-line with the existing axonal trajectory and branching occurred in a orthogonal direction (*D* adapted from [36]).

**Figure 2:**
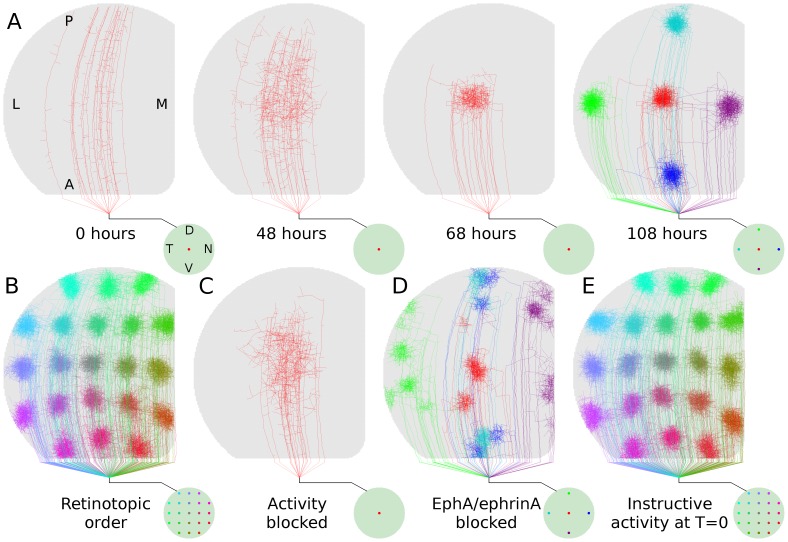
Retinotopic refinement. *A*, Time-lapse development of a group of axons from a specific retinal location. The retina is represented by the circle at lower-right and the colored dot(s) indicate the location of RGCs whose axons are displayed in the colliculus. Development was regulated only by molecular guidance for the first 48 hours whereafter activity-dependent feedback (i.e., regulated trophic release) contributed to simulated development over the next 60 hours. Axon arbors from five retinal locations are shown in the last frame. *B*, Arborizations from 21 points on the retina show retinotopic order in axonal projections. Developmental paradigm is identical to (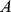). 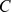, Axon development over 144 hours driven only by molecular guidance. Activity-dependent feedback was required for refinement. 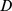, Developmental sequence as in (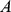), but with molecular guidance blocked along nasal-temporal retinal axis; anterior-posterior collicular axis. Organization is significantly disrupted. 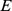, Axon arbor development when activity-dependent feedback contributed to development from the time when axons first began interstitial branching (i.e., T = 0 hr in *A*). Development was qualitatively normal and not dominated by ectopic projections as previously predicted [36], indicating that molecular guidance and activity-dependent mechanisms are able to simultaneously guide and refine development. Data from a single simulation of retinocollicular development are shown in each of (*A*)-(*E*). In all results presented here and elsewhere, three or more simulations were performed (typical runtime 3 days each) and no qualitative differences were observed.

The model is based on a circular retina projecting to an asymmetrical colliculus or tectum (Fig. 1*A*). Simulations began with pioneer axons extended through the target structure and as interstitial branching was beginning, corresponding to development as seen in post-natal day one (P1) mouse and embryonic day ten (E10) chick [41]–[43]. Simulated axons were first guided by only molecular factors for 48 hours producing a coarse arborization in the vicinity of the termination zone (Fig. 2*A*). Unless specified otherwise, all references to time are simulated time. After 48 hours, activity-dependent feedback from spiking postsynaptic cells contributed to axon and synapse development.

First to be examined was how well the model was able to generate normal retinotopic organization. During the first 48 hours, molecular guidance signals allowed axons to generate a course arborization in the vicinity of the retinotopically correct termination zone. After 48 hours, activity-dependent feedback contributed to axon and synapse development. Upon the onset of activity-dependent feedback, arbors quickly remodeled, producing refined projections in the retinotopically correct locations (Fig. 2*A*) and with global retinotopic order (Fig. 2*B*). When activity-dependent feedback was blocked and development was mediated only by molecular guidance, arbors failed to refine even after an extended period of development (144 hours, Fig. 2*C*). When molecular guidance was blocked and organization was mediated only by activity-dependent mechanisms, retinotopic organization was disrupted and extensive ectopic projections were produced (Fig. 2*D*).

The two-stage pattern of growth modeled here, with development mediated by molecular guidance preceding the onset of activity-dependent mechanisms, was based on hypothesized developmental patterns [33], [44] and previous modeling results from GES-2009 [36]. The importance of this assumption was tested by enabling activity-dependent mechanisms from the moment interstitial branching began, allowing both molecular guidance and activity-dependent mechanisms to guide axon growth throughout arbor development and refinement. While this resulted in an increased numbers of ectopic arbors, and sometimes distorted arbors, retinotopic organization and refinement remained qualitatively similar (Fig. 2*E*), generating a behavior inconsistent with the predictions of GES-2009.

**Figure 3:**
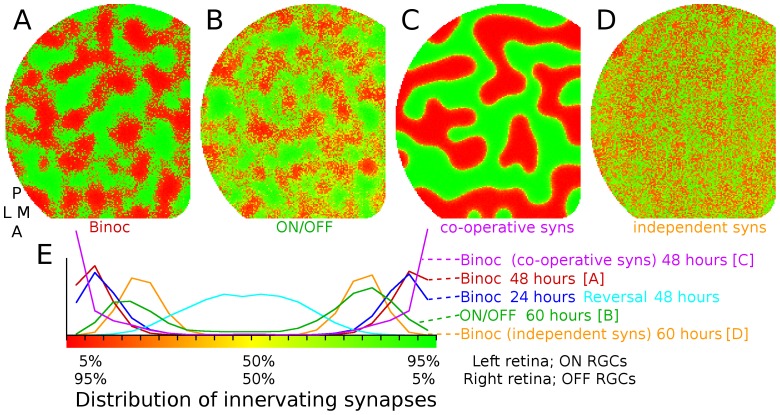
Eye-specific and ON/OFF segregation. 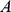
, Spatial segregation of retinal afferents is observed when two retinas with independent patterns of activity project to the same colliculus (tectum). As noted in the main text, the model is based on mouse retinocollicular development but is used generically to explore segregation phenomena observed in other retinal afferent pathways. Colors indicate synaptic drive from either eye (red from left, green from right, yellow equal from both eyes). 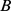, RGCs from a single retina segregate onto different target neurons when RGC activity is spatially correlated but is temporally offset, such as occurs between ON and OFF RGCs during development. 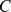, In the present implementation of the model, synapse survival is based both on how much trophic factor is received by the synapse and how much is received by nearby synapses on the axon. This results in synapse survival being a co-operative effort among nearby synapses, but with some degree of independence (arbitrary 75%/25% split). Eliminating this independence so that survival is purely cooperative results in strong spatial segregation. 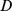, When synapse survival is a purely independent process, such that each synapse's survival depends on how much trophic feedback it receives, segregation occurs but it is not spatially organized. 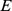, Distribution of segregation under different paradigms. Horizontal axis shows ratio of innervating synapses onto each collicular neuron as a function of retina of origin and vertical axis shows relative number of cells (all normalized to have same area under the curve). The red line shows eye-specific segregation (same data as 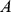) with target neurons becoming highly selective and driven by one or the other retina. When segregation occurs in the binocular paradigm (e.g., *A*) but is followed by a period of development when activity in both retinas becomes synchronized, segregation is reversed (blue lines), as is observed experimentally [34].

There are many examples of afferent segregation in the visual system, with groups of presynaptic cells that have different activity patterns establishing connections to different subsets of target cells. These include eye-specific segregation in cortex [45], superior colliculus [2], [34], [46] and LGN [12], and the segregation of ON and OFF RGCs onto distinct sets of target cells [5], [47]. The paradigm of eye-specific segregation was explored in the model by having axons from two simulated retinas, with independent and uncorrelated patterns of spontaneous activity, project to the same simulated colliculus. Ocular dominance bands were observed (Fig. 3*A, C*), consistent with observations in cortex and retinotectal experiments. Experiments have shown that eye-specific segregation can be reversed if the activity in innervating retinas becomes correlated [48]. This behavior was observed in the model by inducing segregation over 24 hours under the same binocular paradigm as above and then coordinating the retinal wave patterns between both retinas for 48 hours. This eliminated segregation (Fig. 3*E*, dark and light blue lines, respectively).

When different sets of cells in a single simulated retina had spatially correlated but temporally offset activity, similar to what is observed between retinal ON and OFF RGCs during development [37] segregation was also observed (Fig. 3*B*). Specifically, ON and OFF activity was simulated using two groups of RGCs where activity between the groups was spatially correlated but temporally offset, with ON bursts preceding OFF bursts, ON and OFF bursts partially overlapping in time, OFF cells bursting more frequently, and ON cells bursting more intensively. See Methods. A factor underlying spatial segregation in eye-specific and ON-OFF segregation was nearby synapses within an axon helping to stabilize one another, with a strong synapse helping nearby weaker synapses to survive, with the weaker synapses reducing the stability of stronger synapses. When this mechanism was disabled, and each synapse was responsible for its own survival, spatial segregation was eliminated (Fig. 3*D*). Factors affecting patterns of spatial segregation in the model included the width of the dendritic arbor, mechanisms governing synapse stability, spatio-temporal correlation between innervating groups of neurons, the relative activity level between cell groups and the time when Hebbian-based mechanisms first become active (data not shown).

The targeting of RGC axons is mediated by molecular guidance. RGC axon growth in the superior colliculus is guided by countering and orthogonal gradients of guidance molecules, including ephrin ligands and Eph receptors that are both present on RGC axons and on collicular dendrites [23]. These gradients of guidance molecules give each RGC a maximal chemoaffinity with a specific location in the colliculus, providing each axon an approximate target “latitude and longitude” [49] in the colliculus or tectum. Mice bred with mutations affecting the expression of these guidance molecules have characteristic perturbations in development [7], [8], [38]. The ephrinA/EphA family guidance molecules control organization along the collicular anterior-posterior axis, and ephrinA/EphA manipulation is considered here.

**Figure 4:**
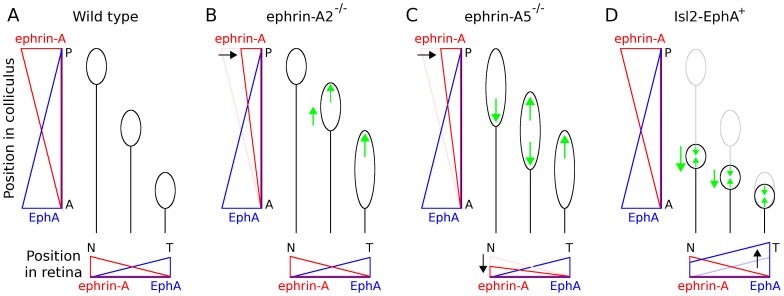
Interpretation of ephrin-A and EphA gradient manipulations. Cartoon depicts chemoaffinity of RGC axons in simulated wild-type and mutant development. Horizontal axes show retinal location, nasal (N) to temporal (T), and the relative magnitude of ephrinA and EphA gradients. Vertical axes show collicular location, anterior (A) to posterior (P), and the relative magnitude of collicular EphA and ephrinA gradients. Circle locations denote the retinotopically correct termination zone and sizes indicate the relative chemospecificity, with larger circles indicating axons having reduced chemospecifity, or chemoaffinity with a broader collicular area. 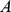, In the simulated wild-type case, all axons are assumed to have similar levels of chemospecificity in retinotopically appropriate areas of the colliculus. 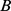, Eliminating ephrinA2 was interpreted as reducing repulsion of RGCs from posterior colliculus, in turn broadening chemoaffinity (reducing chemospecificity) of axons normally repelled from there. 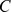, The guidance molecule ephrinA5 is expressed in both posterior colliculus and nasal retina [23]. Its elimination was assumed to reduce chemospecificity of all RGCs, through loss of repulsion to posterior colliculus like in ephrinA2 knock-out, and loss of repulsion from anterior colliculus due reduced repulsion to collicular EphA. 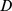, Mutations which upregulate EphA3 in a spatially distributed subset of RGCs [6], [9] results in a single retinal location having RGCs with maximal chemoaffinity for two different collicular locations. Unaltered RGCs have the same preferred targeting as wild-type (gray, as in 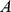) while RGCs with upregulated EphA3 have stronger repulsion from posterior colliculus and are pushed anteriorly (black), forming a second map in anterior colliculus. Heightened repulsion and the compressed map were assumed to increase chemospecificity, as a converse to how ephrinA knockouts reduced it. The model presented here describes the hypothesized functional effect of altering molecular guidance expression. There are many ways that nature might achieve this.

**Figure 5:**
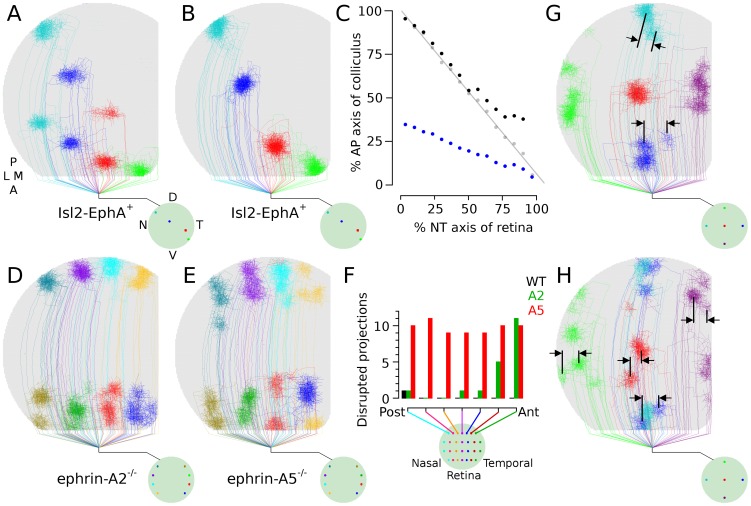
EphA and ephrin-A mutants. 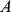
, Arbor development from RGCs at four retinal locations in simulated EphA knock-in experiments (Isl-EphA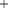), where 50% of RGCs had higher simulated EphA representation and thus stronger repulsion from posterior colliculus. Dual maps are formed. 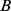, Arbors from the same four retinal locations as in (*A*), but in a control (WT) retina. 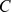, Arborization location in colliculus as function of retinal location. The WT projection (gray) is linear, indicating a single contiguous projection of retina onto colliculus. The EphA projection is split into two contiguous maps, with blue indicating the TZ location of RGCs having upregulated EphA and black showing the locations of unaltered RGCs. Arborizations of unaltered RGCs normally targeting anterior colliculus are shifted posteriorly in simulated mutants (also visible comparing 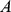 and *B*). 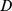, TZ location from eight points in nasal and temporal retina in simulated ephrinA2 knockouts. Arborizations are disrupted in anterior colliculus but are normal in posterior colliculus. 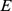, Simulated ephrinA5 knockouts. Organization is disrupted throughout the colliculus. 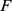, Chart showing relative number of disrupted projections in simulated WT, A2 and A5 retinas, as a function of retinal location. Axons were marked in four locations in seven dorsal-ventral bands of the retina (n = 3 simulations of each type) and the number of arbors having ectopic projections from each band was counted (maximum = 12). While ectopic projections were observed in simulated WT near collicular boundaries, the frequency and degree of these disruptions was minor in comparison to those observed in simulated ephrinA2 and A5 mutants in (*D*) and (*E*). 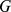,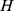 Simulation results from ephrinA5 knock-out (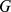) and blocking all ephrinA/EphA mediated guidance (*H*). Manipulating molecular guidance on only the anterior-posterior axis can cause perturbations along the orthogonal axis, a phenomenon observed experimentally [7].

EphrinA/EphA binding generates a repulsive effect in an axon, and the more EphA an axon expresses, the more strongly repelled it is from dendrites expressing ehprinA. RGC axons in temporal retina have the highest EphA expression and are most strongly repelled from posterior colliculus, where levels of collicular ephrinA are highest. Reducing ephrinA expression in the colliculus should thus most strongly affect axons from temporal RGCs by reducing the repulsive force pushing them toward anterior colliculus, in turn reducing their chemospecificity (Fig. 4*B*). Mutations that selectively reduce ephrinA expression in the colliculus (ephrinA2 knock-outs) do result in many ectopic projections being produced by temporal RGCs while organization from nasal RGCs is largely unaffected [7]. In the model, selectively reducing the effect of molecular guidance on temporal RGCs generated similar results, with RGCs that normally targeted anterior colliculus forming abnormal and ectopic projections there while RGCs targeting posterior colliculus were normal (Fig. 5*E*).

Reducing ephrinA expression in both retina and colliculus should affect axons from all RGCs. Axons from temporal retina should be affected as described above, from reduced repulsion to posterior colliculus. Axons from nasal retina, normally high in ephrinA, will be less repelled from anterior colliculus, in turn reducing their chemospecificity (Fig. 4*C*). Experiments which reduced ephrinA expression in retina and colliculus show that ectopic projections occur for both temporal and nasal axons [7]. Running the model with all RGCs having reduced chemospecificity resulted in significant disruptions in targeting and ectopic projections (Fig. 5*F*), consistent with experimental results [7]. Simulations where molecular guidance is blocked along the anterior-posterior axis show much more severe disruption (Fig. 2*D*), consistent with experimental observations that disruptions were more severe when multiple ephrinA guidance molecules were eliminated [7], [50].

When ephrinA/EphA expression is altered, disruption is intuitively only expected along the collicular anterior-posterior (A-P) axis, as these molecules are expressed in gradients along this axis. Ectopic projections and disruptions are still observed along the medial-lateral (M-L) axis, however [7], [8], despite there being no known changes to molecular guidance along this axis. In the model, disruption of molecular guidance along only the collicular anterior-posterior axis resulted in ectopic and disrupted axonal projections along the medial-lateral axis (Fig. 5*G, H*) consistent with experimental data (e.g., [7]).

When EphA is upregulated in a spatially distributed subset of RGCs, axons from a single retinal location form ordered projections in two collicular locations, producing a dual map [6], [9]. The upregulation of EphA on an axon is here interpreted as increasing its repulsion from areas of high dendritic ephrinA expression. This results in the “retinotopically correct” termination zones being pushed toward anterior colliculus and chemospecificity increased, due to map compression (Fig. 4*D*). Running the model based on this interpretation resulted in axons from a single retinal location producing dual maps in the colliculus (Fig. 5*A*) with the map created by unaltered RGCs being slightly compressed and pushed posteriorly (Fig. 5*C*), phenomena that are both observed experimentally [6], [9].

## Discussion

The present model and its structurally related predecessor GES-2009 addressed the network-level phenomenon of retinotopic organization as being an emergent property of many causative and contributing cellular-level behaviors, including axon and synapse growth and retraction. The present study also examined how the interactions of these factors could generate segregation and reproduce developmental perturbations that result from changes to the expression levels of guidance molecules.

The present model is structurally and functionally similar to GES-2009 and retinotopic organization occurs in a similar way. The outline of the events generating a refined retinotopic projection are:

1. axons produce coarse arborizations in the vicinity of their retinotopically correct termination zones (RC-TZs). This results in nearby RGCs having arbors that largely overlap, with the highest density being near the RC-TZ;
2. Synapse formation is restricted to locations where axons are present. Because the highest concentration of axons from a particular retinal location is near the RC-TZ, and because chemoaffinity is highest there, the highest density of synapses from that retinal location is also there;
3. Spatio-temporally correlated retinal activity (‘retinal waves’) causes nearby RGCs to fire together, resulting in the collicular (tectal) neurons near the RC-TZ of these RGCs also firing, due to the relatively high synaptic density from this part of the retina. This results in trophic feedback to the synapses contributing to collicular cell firing;
4. For a given RGC, synapses near the RC-TZ are more likely to induce a postsynaptic spike and receive trophic feedback than synapses farther away, and hence synapses farther away were more likely to retract;
5. Trophic feedback delivered preferentially to synapses near the RC-TZ results in increased axon and synapse growth near the RC-TZ, further increasing the synapse density there and producing a self-reinforcing process that caused the arbors to refine at the RC-TZ.

All of these behaviors were required to reliably generate retinotopic organization. Perturbing molecular guidance disrupts (1) and thus (2), eliminating proper targeting (e.g., Fig. 2*D*) or disrupting it (Fig. 5), depending on the degree of perturbation. Even when guidance was perturbed, the remaining behaviors (3–5) allowed axons from nearby RGCs to refine their arbors, but not necessarily in the correct locations. Blocking correlated activity disrupts (3) and thus (4), allowing axons to be properly targeted but unable to refine (Fig. 2*C*). Disrupting trophic feedback (5) is equivalent to blocking (3) and (4), as trophic feedback is the mechanism through which activity-dependent guidance is mediated.

Eye-specific segregation is thought to occur through Hebbian processes, with cells having temporally uncorrelated activity patterns segregating to innervate different groups of target cells, although there is circumstantial evidence for the involvement of eye-specific molecular markers [51]. In the model, segregation of afferents onto different sets of target cells occurs because synaptic stabilization produces a Hebbian-based positive feedback mechanism, with a target cell responding preferentially to one retina strengthening the synaptic connectivity from cells of the same retina and weakening the synapses from the other. Spatial patterns of segregation are influenced by two factors in the model, both regulated autonomously within each axon. The first is the degree that nearby synapses on an axon help to stabilize one another. Co-operating synapses help to prevent retraction of nearby synapses that project to target cells dominated by input from the opposite eye, helping to change which eye the target cell responds to input from, thereby influencing spatial patterns of segregation. The second is that axons are more likely to branch, stabilize and generate synapses in sections of the axon having stronger trophic feedback to synapses, which occurs when synapses are more successful at inducing spikes in their targets. Both mechanisms generate a positive-feedback mechanism which helps to induce spatial segregation. Consistent with model assumptions about axon autonomous signaling, experimentally it has been shown that nearby axonal synapses share presynaptic vesicles and proteins during development [52], providing a way for synapses to share molecular resources and assist each other, and synapse and axon growth are both associated with trophic factor receipt [15], [17], providing a mechanism to enhance growth in parts of an axon where synapses receive more trophic support.

**Figure 6:**
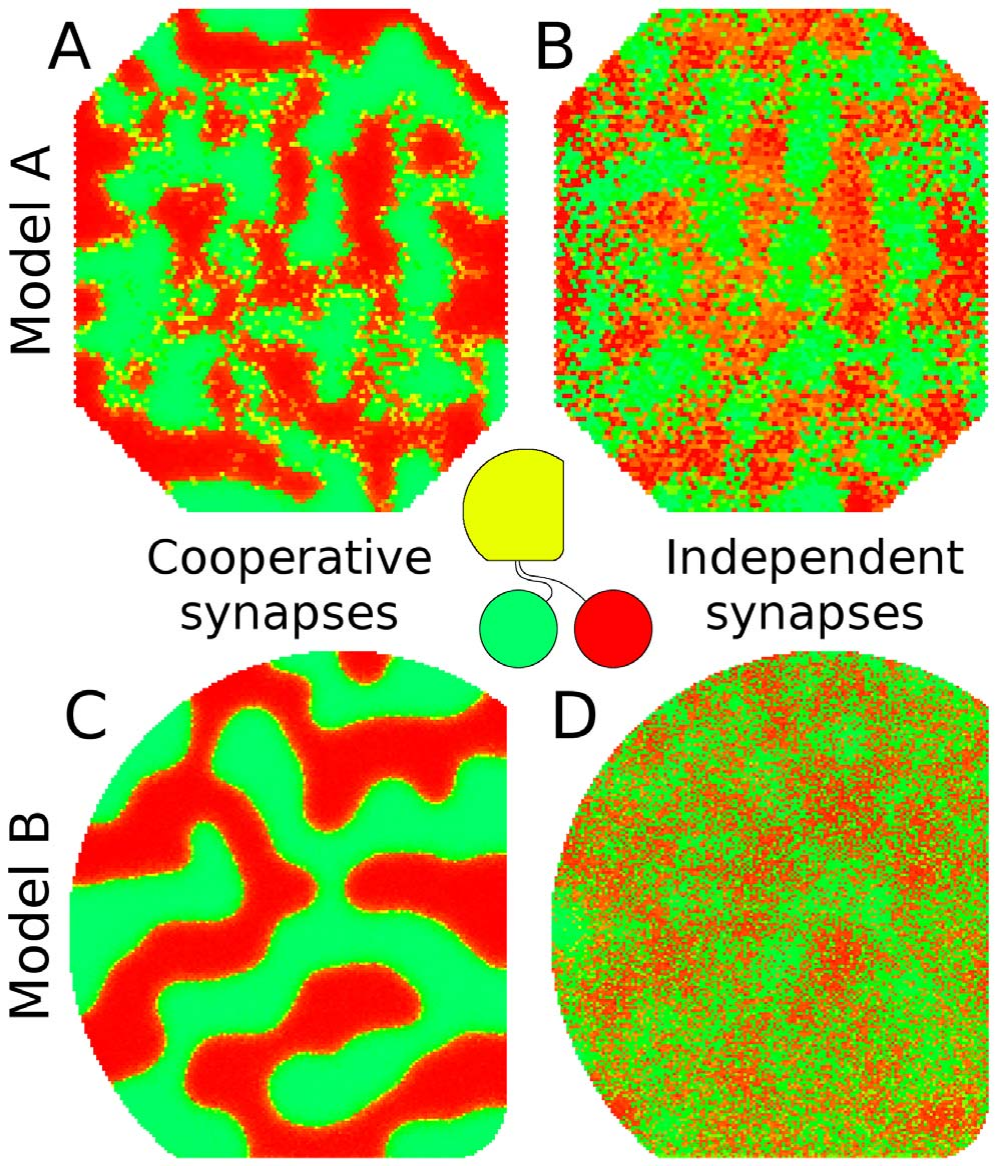
Different mathematical models of the same general description and structure give rise to different behaviors. In early visual processing, visually responsive neurons in the midbrain and cortex typically become selective to input from a single retina (e.g., [34], [45], [46]). In the center of the image is a cartoon showing axons from each retina targeting the same midbrain structure, in this case a simulated superior colliculus. Collicular neurons are color-coded to show which retina they receive their primary input from (green is left retina, red is right, and yellow is equal from both). 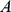, When the previous model (GES-2009, [36]) was modified to run in this binocular paradigm, it exhibited segregation when two simulated retinas projected to the same target structure. 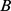, When eliminating a model assumption affecting synapse stability, segregation was perturbed very little. This assumption was that the stability of a synapse was influenced by the stability of other nearby synapses on an axon – i.e., synapse survival was co-operative or synapses were independent. 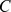, The model in the present study similarly exhibited segregation. 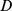, When the same assumption was dropped in the model in this study, the characteristics of segregation changed significantly. This observation in the present study leads to the prediction that altering synapse stability mechanisms can influence patterns of spatial segregation, although this prediction is not fully supported by GES-2009.

The results of the model suggest that cooperation between synapses and factors influencing their survival are dominant factors governing spatial patterns of segregation (Fig. 3) and a prediction from the model is that altering the mechanism governing synapse survival will alter spatial segregation – spatial order will be strong when nearby synapses on an axon support each other and order will be weak or absent when synapses are independent (Fig. 6*C, D*). To test this prediction, the model of GES-2009 was modified by adding a second retina. This modified GES-2009 model was tested to verify that it exhibited eye-specific segregation and to explore the effects of altering the synapse survival mechanism. Spatial segregation was indeed observed but eliminating the mechanism whereby nearby synapses supported each other had only minor effects (Fig. 6*A, B*).

During development, retinal ON and OFF cells have spatially correlated but temporally offset spontaneous firing patterns [37]. As with eye-specific segregation, this temporally separate pattern of activity was sufficient to generate segregation in the model. ON and OFF cells also have different activity profiles, with OFF cells bursting more frequently and ON cells bursting more strongly. The relative intensity of activity was not found to be important for segregation itself but it did influence the relative number of target cells recruited by ON and OFF RGCs. The results demonstrated here relating to ON-OFF segregation run contrary to a recent spike-based model that concluded ON-OFF segregation was not possible based only on the spiking patterns of individual RGCs [53]. The likely reason for this discrepancy is that only a single target cell was used in that model [53], preventing groups of ON and OFF cells from having different targets to segregate onto.

The model was able to qualitatively reproduce many aspects of the effects of up- and down-regulation of ephrinA and EphA guidance molecules. The reason for the simulated disruption parallels the reason for disruption when molecular guidance is blocked. When the simulated role of guidance molecules is blocked, axons are forced to target and refine based only on activity-dependent mechanisms, resulting in globally disordered projections. When guidance molecule expression is present but reduced, some information is available to help guide axons, reducing the degree of global disorder. When altered expression is restricted to a subset of the colliculus, or the afferents from a subset of the retina, abnormal or disordered projections occur in a subset of the colliculus.

**Figure 7:**
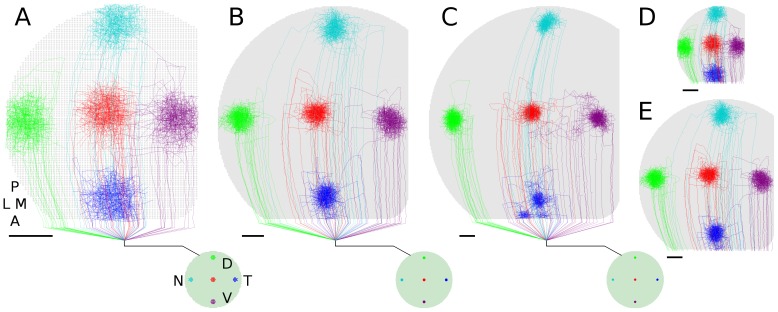
Arborization size relative to collicular area. Relative size of axon arbors to collicular area changes with colliculus size when collicular cell density is held constant (scale bars = 100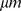). All colliculi scaled to same display size to show relative arbor coverage. Colliculi have 6772 cells (*A*, 2515 RGCs), 25K cells (*B*, 10K RGCs) and 50K cells (*C*, 20K RGCs). All results shown are based on the same simulation parameters, other than number of RGCs and collicular cells. 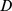,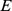, Same colliculi in (*A*) and (*B*), respectively, shown to same scale of (*C*). Simulation results can thus be influenced by the number of neurons represented, indicating that the scale of a computational model can influence its quantitative results.

The disruptions occurring in simulated ephrinA knockouts (Fig. 5*D, E*) were more modest than are observed experimentally [7], [54]. One possible reason for this is that the simulated changes to chemoaffinity and chemospecificity were less than what is induced experimentally. Another reason is due to scaling factors, as the size of the modeled colliculus is smaller than mouse colliculus where experimental observations were made (smaller meaning having less target cells and lower overall dimension). The size of simulated axon arbors relative to collicular area changes with collicular size, holding the density of collicular cells constant (Fig. 7) and axon dynamics can be expected to change based on how much area an axon covers and its degree of overlap with other axons. This reasoning suggests that repetition of ephrinA knock-out experiments in chick, whose tecta are smaller than mouse colliculi while developmental patterns are similar [23], should produce more modest disruptions than are observed in mouse. Results from the present model indicate that results can be scale dependent in scaled computational models, so in future modeling studies it might be useful and insightful to run full-sized simulations, as results would not be subject to scaling factors and a better quantitative fit to experimental data could be pursued. Other modeling fields have mechanisms to compensate for changes in scale (e.g., the Reynolds number in fluid mechanics), but neural modeling is a much larger and varied field and as yet it lacks established and mathematically justifiable ways to compensate for scaling factors, potentially reducing the theoretical utility of quantitatively accurate models that are of reduced scale.

In the case of simulated EphA upregulation (Fig. 5*A, C*; [6], [9]), approximately half of RGCs had their TZs shifted anteriorly, with these RGCs being randomly scattered about the retina. This effectively produced overlapping maps in anterior colliculus, in turn increasing competition for the collicular neurons there. When arbors refined, they did so in a way consistent with the two maps, based on the same behavior described above for normal retinotopic organization. The increased competition in anterior colliculus shifted the arbors of unaltered RGCs posteriorly, where competition for target cells was less. Both dual maps and the shifting of arbors from unaffected RGCs are observed experimentally [6], [9]. As with modeled ephrin mutants, pursuit of quantitative matching to experimental data in modeled EphA mutants will require a full-scale model of the colliculus, as model arbor dimensions do not scale linearly with colliculus size (Fig. 7).

The model presented here is similar in description and structure to GES-2009 and it has been applied to a wider range of phenomena than the previous model. These phenomena include ocular dominance, the segregation between ON and OFF retinal afferents, and perturbed retinotopic development where ephrinA and EphA guidance molecules are knocked-out or upregulated. A number of changes were made to the current model which involved changing the mathematical implementation of represented behaviors (e.g., axon growth rules; synapse formation and stability; etc.) while maintaining the qualitative properties of those behaviors. These changes were made in order to explore the effects of choosing different mathematical implementations of the same general behavioral descriptions.

Different neural pathways within and between species exhibit different organizational properties, and similarly, there are differences in cellular behaviors, developmental periods and patterns of spontaneous activity between species and different brain areas. It might be expected that the mathematical differences between the present model and GES-2009 would also result in differences in model behaviors, and this was indeed the case. The differences were relatively subtle, similar to those that might exist between species, although they did yield different predictions. The present study contradicting a prediction of GES-2009, which was that if activity-dependent mechanisms were active before axons had established coarse arbors near their TZ then an orderly retinotopic map would be disrupted (compare Fig. 2*E* to Fig. 8 in [36]). A prediction from the present study, that altering the mechanism governing synapse survival will alter spatial segregation, was not supported by GES-2009 (Fig. 6). It is possible that the predictions and model behaviors reported here and in GES-2009 are each valid but in different retinal pathways, different species and/or different genetic strains within a species, but at present there is insufficient data to indicate whether or not this is the case.

Biology is often too variable and complex to be usefully represented by simple equations and sometimes biological observations and hypotheses are not easily translatable into precise mathematics. However, many modelers consider the conversion of biological hypotheses to precise mathematical form to be a benefit as it allows identification of hidden assumptions in descriptive models [55]. It is rarely considered that several mathematical forms may be consistent with a given descriptive hypothesis and that the output of a model, including its insights and predictions, can depend on the choices, or implicit assumptions, made when performing this conversion. Exploring the behaviors of different mathematical implementations consistent with a verbal description of a model may usefully yield different results. If a model is robust to such variations it implies that the constraints on the underlying biology are relatively weak and/or well understood and that further experimental investigations may not be particularly revealing. Differences between model outputs might point to behaviors that would benefit from more detailed experimental and descriptive characterization. In the present study, retinotopic organization appeared to be robust to the various mathematical implementations that were explored. Other behaviors, such as the degree and extent of spatial segregation and activity-dependent mechanisms of axonal guidance appeared more sensitive to implementation details. The behaviors underlying axonal branch formation and retraction, as well as synapse stability, are likely candidates for describing why the present model and GES-2009 exhibited different behaviors and predictions. As yet we are unable to explain or demonstrate why this might be so.

This study demonstrates how a broad range of network-level experimental observations can be accounted for by rules that govern the growth and retraction of axons and synapses. The model is able to account for the development of refined retinotopically organized projections, eye-specific segregation, segregation of ON and OFF RGC afferents, and perturbed retinotopic development where the ephrinA and EphA guidance molecules are knocked-out or upregulated. The model explicitly represents a broad range of processes/mechanisms underlying development, ranging from molecular guidance cues to synaptic plasticity resulting from individual neural spikes. By directly representing these factors, in particular the branching and retraction of individual axon arbors, the model has a level of detail that can be better compared to what is observed experimentally. The cellular behaviors and mechanisms represented, such as axon and synapse growth dynamics, correlated patterns of spontaneous activity, ephrin guidance markers and growth factors are observed in many areas of the developing nervous system and in many different species, so the model and results presented here may be adaptable to neural development more generally.

## Methods

**Figure 8:**
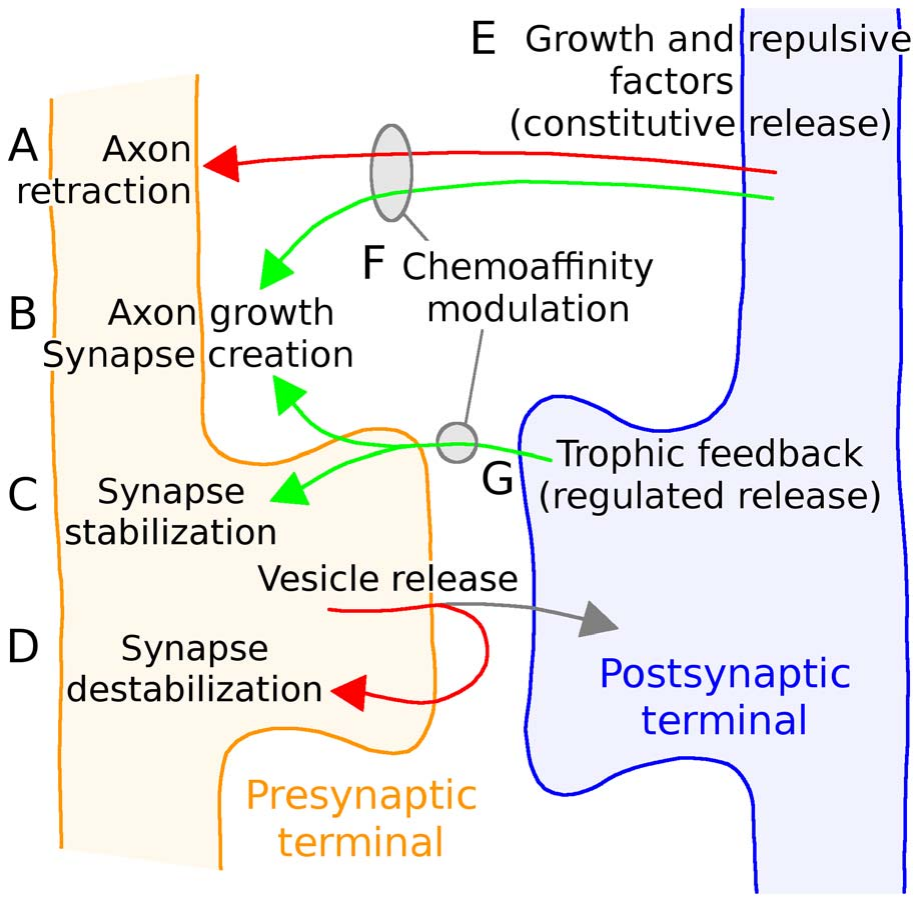
Description of model structure. The behaviors described in Fig. 1*B* are implemented through different model equations. 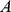 Axon retraction was governed by the relative concentrations of growth and repulsive factors (Eq. 11). 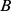 Axon growth was influenced both by the relative concentrations of growth and repulsive factors and by the total arbor size (Eq. 10). Synapse formation was influenced by the axon's local exposure to trophic factor and the number of existing axonal synapses (Eq. 13) and the target cell's firing rate and total number of dendritic synapses (Eq. 14). 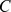,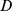 Synapse survival was governed by the total amount of trophic factor received by the synapse, the number of existing axonal synapses, the firing rate of the postsynaptic cell, and optionally the trophic factor received by neighboring axonal and dendritic neighbors (Eqs. 16,17) 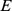 Growth and trophic factors were continuously released into the extracellular space and diffused locally (Eqs. 6,7), based on each cell's firing rate and it's relative maturity (Eq. 3). 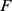 The chemoaffinity and chemospecificity modulating axon and synapse growth was based on how far an axon segment was to its retinotopically correct termination zone (Eq. 2). 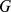 Trophic feedback was provided to the presynaptic terminal when a spike in the postsynaptic cell followed a spike in the presynaptic cell within tens of milliseconds (Eq. 12). The activity of simulated RGCs was governed by a phenomenological model of retinal wave activity [64] and postsynaptic neural activity was based on an integrate and fire neuron (Eqs. 4,5).

An overview of the model is presented here. The mathematical description is provided below and Fig. 8 describes how model behaviors map to the equations. Simulation source code is available upon request.

RGCs from a simulated retina of 10 K cells each extended axons into a simulated colliculus of 25 K cells having an inter-cellular spacing of 6 *µm* and dendritic arbors 50 *µm* in diameter, yielding significant overlap between dendrites of adjacent cells. Axons were composed of segments, each 11 *µ*m in length, and each segment was able to extend, branch and retract (Fig. 1*D*). In binocular simulations, two retinas (10 K cells each) projected to the same colliculus. For computational convenience, the soma of each collicular neuron was positioned in the center of its dendritic field. The tight inter-cellular spacing of collicular neurons, relative to soma diameter, was assumed to be achieved through vertical offset of the somas (Fig. 1B).

Collicular neurons were integrate and fire neurons and each had a target firing rate (0.5 Hz). To help maintain this rate, existing synaptic connectivity was modulated both by changing the strength of synaptic excitation [35] and by influencing the relative stability of innervating synapses by up- and down-regulating trophic feedback. To attract new input, immature neurons and neurons that were firing below their target rates diffusively released growth-inducing factors (e.g., BDNF). Factors inhibiting growth (e.g., proBDNF) were released by mature neurons and neurons that were over-active.

The behavior of simulated neurons changed over time to simulate maturation of the cells. As model neurons matured, they increased their electrical size (e.g., conductance and capacitance), affecting the somatic EPSP of synaptic inputs, with a single synaptic input received on a mature neuron inducing a smaller EPSP than an input on an immature neuron. Simulated collicular neurons also constitutively (continuously) released growth and repulsive factors into the extracellular space to regulate the attraction of axons and synaptic input. Immature neurons released more growth-inducing factors and fewer growth-repulsive factors than mature neurons.

Biologically, molecular guidance of retinal axons is provided through bidirectional signaling between ephrin ligands and Eph receptors that are on both retinal axons and collicular dendrites, and these molecules co-localize and interact with the trophic factor receptors for BDNF and proBDNF [55]–[59] and each other [60] as well as share intracellular signaling pathways [61]. The implementation of constitutive BDNF and proBDNF release, and the molecular guidance provided by gradients of Eph and ephrins, were thus combined, with Eph and ephrins regulating the relative efficacy of receptors for BDNF and proBDNF. Specifically, the closer an axon segment was to its retinotopically correct area of the colliculus, the higher its chemoaffinity, and hence the higher the efficacy of its response to growth-inducing factors, increasing the probability of axon and synapse growth. Similarly, the further an axon segment was from its target area in the colliculus, the weaker its chemoaffinity and the more sensitive it was to repulsive factors.

Simulated RGC axons extended into the colliculus, with patterns of axon growth following descriptions of growth as reported in mouse and chick [22], [23], [62]. In summary, simulations began with each RGC axon extended along the anterior-posterior (A-P) axis of the colliculus and before significant interstitial branching had occurred, corresponding to development as seen in P1 mouse and E10 chick [41]–[43]. The position of each axon along the lateral-medial (L-M) axis was near the L-M position of its retinotopically correct termination zone [38], [42]. Except as otherwise specified, each axon was shifted 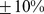 of the width of the SC from this position (% as standard deviation; Gaussian distribution). In order to allow axons to produce a largely uniform coverage of the asymmetrical colliculus, each axon had a curved trajectory to help it maintain its relative L-M position throughout the colliculus. Axon extension and branching occurred probabilistically based on the segment's chemoaffinity, the presence of constitutively released growth factors, and the trophic feedback to the synapses on the axon segment. Each 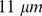 segment could support up to three synapses. Retraction occurred only at axon tips and was based on the ‘chemorepulsion’ of the segment (the inverse of its chemoaffinity) and the presence of constitutively released repulsive factors. Axon growth was structurally similar to GES-2009, with the only difference being the size of the segments (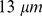 versus 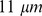, see Fig. 10*D* in [36]).

Axon arborization occurred in two stages, with initial growth driven by the simulated effects of molecular cues, guiding each axon to near its retinotopically correct termination zone. Once axons had reached near to their termination zone, activity-dependent mechanisms contributed to arbor refinement, consistent with experimental descriptions [33], [44], [63] and the same as previous theoretical work [36]. Axons growth occurred under guidance of molecular guidance for the first 48 hours. Unless otherwise specified, all references to time are simulated time. After 48 hours, activity-dependent mechanisms also played an instructive role and development continued for another 60 hours. In some simulations, activity-dependent mechanisms were activated from the beginning, when axons first began their interstitial branching, and these simulations ran for 72 hours. When eye-specific segregation was addressed, the activity-dependent period lasted only 48 hours (unless otherwise specified) as segregation occurred quickly. The simulation timestep was 1 ms. The parameters governing axon and synapse growth and retraction were selected to produce development occurring on this time scale, but no efforts were made to fine tune the parameters. As with GES-2009, a broad range of parameters was able to produce qualitatively similar outputs. The systematic examination of parameter values performed in GES-2009 was not performed here, due to the time required for each simulation, but there was nothing to indicate that the present model would not exhibit similar stability qualities.

Molecular guidance of retinal axons was implemented as each axon having an approximate target “latitude and longitude” in the colliculus [49]. Segments of an axon that were closer to this target location had a higher chemoaffinity for their surroundings than segments of an axon farther away. Countering gradients of guidance molecules [23] were not explicitly represented, only their hypothesized net effect (Fig. 4). Decreased expression of molecular guidance markers, such as occurs in ephrinA knockouts [7], was hypothesized to result in reduced chemospecificity for axons affected by the specific ephrinA molecule that was knocked-out. Increased expression of guidance molecules, such as selective EphA knock-in [6], , resulted in affected axons having their termination zones shifted anteriorly and away from areas of higher ephrinA expression, and their chemospecificity was increased.

Synapse formation was based on the same factors regulating axon extension and branching. Synapses were formed between axon segments and collicular neurons whose dendritic arbor (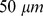 diameter) overlapped the segment (i.e., synapses could form with collicular neurons that were up to 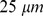 away). Synapse generation was only successful if the target neuron accepted the new synapse. Factors regulating synapse acceptance were the firing rate of the collicular neuron, as over-active cells were less likely to accept a synapse than under-active ones, and the existing number of existing synapses from the axon's RGC, as each collicular neuron was restricted to obtaining a maximum amount of input from any individual RGC.

Synapses operated based on a mechanism consistent with the synaptotrophic hypothesis [16], requiring trophic feedback to survive. Trophic feedback was supplied to a synapse when a spike in the postsynaptic neuron occurred shortly after a spike in the presynaptic neuron. Synapses on an axon that received less than the average amount of trophic feedback, compared to other synapses on the axon, were subject to retraction, with the worst performers being most likely to retract. Similarly, synapses on a dendrite that received less than the average amount of tropic feedback compared to other synapses on the dendrite were subject to retraction. All synapses had unitary synaptic weights and were not subject to plasticity affecting their EPSPs, based on previous results [36]. Plasticity was realized through the creation and activity-dependent retraction of individual synapses.

Activity in RGCs was driven using a phenomenological model of retinal waves [64] that was modified to support simulated bursting activity of ON and OFF RGCs [37]. Simulated retinal waves were generated having an average velocity of 130 *µ*m/sec, an average inter-wave interval of 90 seconds, and wave sizes averaging approx. 0.95 mm^2^. RGC bursts were at 20 Hz with a duration of 2.5 sec, for an overall firing rate of 0.5 Hz, comparable to observed values [65], [66]. Retinal wave activity was pre-computed on a 22 K RGC retina and the same wave activity was used for all monocular simulations. Binocular simulations used two such retinas, each having the same wave statistics but distinct wave patterns. In simulations addressing ON/OFF segregation, the spatio-temporal properties of waves remained the same but the bursting dynamics of RGCs was altered, with ON cells participating in only 67% of waves while OFF cells participated in all waves, and with ON cells preceding OFF cells by one second when they did burst. Each wave generated three cycles of bursting activity, coarsely approximating experimental observations, and the burst rate for ON and OFF cells was 24 Hz and 20 Hz, respectively, based on the asymmetric patterns of firing rates observed experimentally [37]. Burst duration for ON and OFF cells was 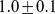 seconds.

The model was implemented in multi-threaded C++ and simulation data were saved in an embedded database (sqlite). Data analysis was performed on the database and simulations could be resumed based on data stored there. Simulation source code and analysis tools are available upon request. Simulation runtime was typically 3 days on a 6-core XEON CPU workstation or cluster node and all simulations were run at least three times from independent starting points. All results were qualitatively similar.

### Comparison between GES-2009 [36] and the present model

Both models were structurally similar and simulated retinocollicular development as is observed in mouse and chick, and the causative and contributing factors are similar between models. Both were based on the hypothesis that axon and synapse growth was most likely in parts of an axon that had higher amounts of trophic feedback and higher chemoaffinity with the surrounding tissue and that synapses required trophic feedback to survive. Structurally, both were based on the growth and retraction of individual axons and synapses and the represented behaviors had similar functional roles. Each was purposefully implemented in a mechanistically and mathematically distinct way, however, both to simulate the case where two different modelers each created models based on the same hypothesis and to examine the robustness of results. The behaviors represented in each model included:

#### Axon growth

Simulated axons consisted of segments of axons that each could extend, branch and retract. These events occurred based on the presence of growth and/or trophic factors and the chemoaffinity of that part of the axon with surrounding tissue. Axon growth and retraction was regulated through a threshold and resource-based mechanism (GES-2009) or occurred probabilistically based on factors affecting each segment (present model).

#### Growth and trophic factors

Synapses received trophic feedback when the postsynaptic cell fired within tens of milliseconds of the presynaptic cell. Receipt of trophic factor reinforced the synapse and increased the likelihood of synapse and axon growth in the vicinity of the synapse. Trophic feedback provided a positive reinforcement signal (GES-2009) or attractive and repulsive signals whose ratio depended on the interval between pre- and postsynaptic spikes (present model). In the present model, postsynaptic neurons were assumed to release a growth factor to attract synaptic input to “immature” and under-innervated cells, a behavior that was examined but disabled in GES-2009 due to its minimal influence on development in that model.

#### Molecular guidance cues

Each axon had an area of the colliculus (tectum) where it had a maximal affinity for growth that was based on the relative location of the axon's soma in the retina. This affinity influenced the relative growth and retraction of axon segments and the formation of synapses. Chemoaffinity was explicitly calculated based on an approximation of the interactions and concentrations of gradient molecules (GES-2009) or the algorithmically equivalent net effect of these interactions was approximated, with chemoaffinity being maximal at the retinotopically correct termination zone and falling with distance (present model). In the present model, there was also an association between chemoaffinity and trophic and growth factors, with chemoaffinity modulating their efficacy.

#### Synapse dynamics

Synapses were established between axon segments and nearby dendrites of collicular cells and were regulated by both pre- and postsynaptic cells. On the presynaptic side, synapse formation was based on the same mechanisms as axon growth. On the postsynaptic side it was influenced by the activity level of the postsynaptic neuron and the existing number of synapses between the two cells. Synapses were reinforced through simulated trophic factor receipt and synapses on an axon helped to stabilize or reinforce other synapses on nearby axon segments (i.e., synapse survival was co-operative). Synapse retraction was based on a resource-based mechanisms within the axon (GES-2009) or by synapses probabilistically retracting when they received less than the average amount of trophic feedback relative to other synapses on the axon or dendrite (present model).

#### Hebbian plasticity

Hebbian plasticity was realized through the creation and retraction of synapses. Spike-timing dependent plasticity (STDP) was implemented previously (GES-2009) where it was shown that the distribution of synaptic weights was near unity (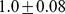) and that synapse-specific plasticity was not necessary for retinocollicular development. Based on these results, the plasticity of individual synaptic weights was not represented in the present model.

#### Spontaneous retinal activity

RGC activity was driven by a phenomenological model of retinal waves [64] that produced patterns of activity which were more strongly correlated between nearby neurons than neurons further apart. The spatio-temporal properties of this activity were varied broadly without producing significant changes in retinotopic organization (GES-2009) or activity was generated with a single spatiotemporal profile (i.e., same wave size, frequency, duration, etc.) and where subsets of neurons had activity that was temporally offset in some simulations (present model).

#### Model Neurons

Integrate-and-fire neurons were used that matured over development, with this being realized by changes to the electrical size of the cells. The change in size was monotonic (GES-2009) or could be reversed if the cell's firing rate became too low, such as if shrinking due to insufficient synaptic input (present model).

#### Homeostatic controls

Collicular neurons up- and downregulated the strength of innervating synapses and altered the cell's willingness to accept additional synaptic inputs based on its firing rate relative to a target rate. The growth and branching of axon segments, and the establishment and stability of synapses, were also influenced by the number of axon segments or synapses present in an arbor, respectively. Collicular neurons down-regulated trophic feedback to innervating synapses with increased firing rate to help limit total innervation, effectively creating a form of presynaptic competition for postsynaptic resources. The equations implementing these behaviors differed between models, sometimes because changes to how model elements interacted required this (e.g., axon segments) and other times to provide a similar equation having different mathematical properties (e.g., exponent change in postsynaptic regulation of trophic feedback).

### Mathematical description

Several formulas in the model utilize a sigmoid-like function that has a stable, near-unity value for small 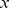 and that decays to zero with increasing 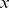. The following family of functions was used for these cases:
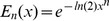
(1)


This function has the value 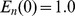 and 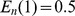 for all positive 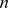. The flatness of 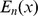 for low 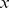, and the steepness of its decay, varies with 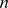. Square brackets in equations were used to indicate when a term in an equation has a lower bound, which is indicated by the subscript following the brackets. For example, 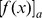 means that 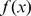 has a lower bound of 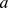. The use of ‘
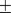
’ signifies the addition of a random number with Gaussian distribution.

The biological mechanisms represented here (e.g., axon growth and branching; synapse formation and retraction; synapse stability; homeostatic mechanisms; molecular guidance; etc) have similar behavioral properties to those of previous implementations of the model [36, unpublished] but are mathematically distinct. There are many design decisions that must be made about how the different behaviors represented in a model of neural development are implemented, even models based on relatively simple concepts such as the molecular induction hypothesis [67], [68] or other models addressing topography and segregation in the visual system [69]. The present model was designed to be mathematically distinct form of the same hypotheses, structure and mechanisms of previous models in order to better analyze the stability of results coming from a model based on the same hypothesis and represented behaviors.

#### Molecular guidance cues

Each RGC was assumed to have a retinotopically correct termination zone (RC-TZ) in the colliculus that was identified by molecular markers, with the relative chemoaffinity of each section of an axon being a function of its distance from the RC-TZ of its parent RGC. The chemoaffinity modulating growth, 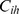, of axon segment 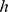 of RGC 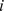, was:
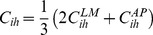
(2)where 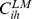 and 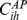 were the relative strengths of molecular guidance along LM and AP axes, respectively, with 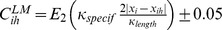 and 
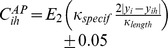
. In these equations, 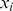 and 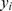 were the coordinates of the RC-TZ for RGC 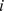, 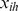 and 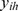 were the coordinates of the axon segment, 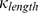 was the length of the colliculus, 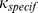 was the relative chemospecificity (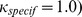 and 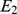 was a sigmoid-like function (Eq. 1). The net result of these equations was that 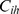 was highest near an axons retinotopically correct target in the colliculus and it decayed with distance from this point. The specificity of targeting governed the rate of decay with distance. The stronger emphasis on response to the LM gradient was found to be necessary to produce phenomenological patterns of axon growth along the LM axis.

In simulations addressing ephrinA mutations, the specificity of the chemoaffinity signal was reduced in RGCs affected by changed gradients (Fig. 4*B, C*). For ephrinA2 mutants, 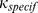 remained unchanged in axons targeting anterior colliculus and reduced linearly to 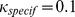 for those targeting posterior colliculus. For ephrinA5 mutants, 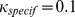 for all RGC axons. In simulations addressing EphA mutations, RGCs having upregulated EphA had their TZ mapped onto the anterior third of the colliculus and chemospecificity was increased 3-fold (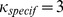).

#### Model Neurons

Collicular neurons were integrate and fire neurons that were considered to mature over the course of development. Maturation included increase in electrical size, affecting the somatic EPSP of synaptic inputs, and changing the amount of trophic factor released. The size, 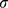, of collicular neuron 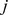 was:
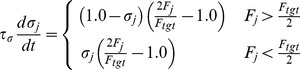
(3)where 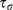 was the time constant of growth (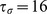 hours), 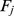 was the firing rate, as estimated over a moving 20 minute interval, and 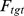 was the target firing rate (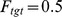 Hz). An immature neuron had 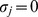 and a mature neuron had 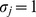.

The excitation level, 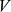, of neuron 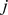 was:
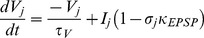
(4)where 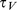 was the time constant for excitation (analogous to the membrane time constant; 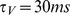), 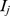 was the present level of synaptic input (Eq. 5), 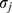 was the relative size of the neuron (Eq. 3), and 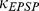 was a scale factor governing the strength of synaptic input measured at the soma as a function of neuron size (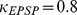). When 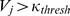 (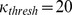), the neuron was considered to generate an action potential and 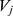 was reset to zero. The variable 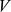 represents summed synaptic input and does not directly represent voltage.

Synaptic input 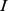 to neuron 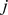 was:
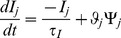
(5)where 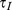 was the time constant for excitatory synaptic input (AMPA receptors, 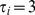 ms, e.g., [70]), 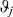 was the homeostatic scaling factor that regulated synaptic strength as a function of the firing rate of the cell (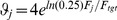) and 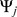 was the number of innervating synapses on 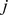 that “fired” (i.e., released a vesicle) in the previous millisecond. Based on the modeling results and synapse plasticity arguments in [36], all synapses had a unitary strength and the plasticity of individual synapses was not represented. Regulation of the strength of innervating synapses was to help control the firing rate of the neuron [35].

#### Extracellular space

The colliculus was partitioned into a grid of 10×10 *µ*m squares which were used to store the concentration of substances released by collicular neurons into the extracellular space. The concentration of each compound was considered to be constant throughout the grid square and the diffusion of these substances was limited to the area in the colliculus occupied by the cell's dendritic arbor (dendrite radius  = 25 *µ*m).

Immature neurons and neurons below their target level of activity were considered to constitutively release a growth-inducing factor (e.g., BDNF) that induced local axon growth and synapse generation, and a repulsive factor (e.g., proBDNF) when mature and above their target activity level in order to inhibit local growth. The concentration of extracellular growth and repulsive factors decayed exponentially. The concentration of BDNF, 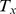, was:
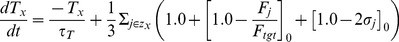
(6)where 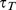 was the time constant governing growth factor decay (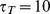 min), 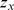 was the set of all collicular neurons having part of their dendrites in grid unit 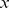, 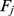 and 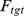 were the current and target firing rates of collicular neuron 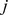, respectively, and 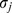 was the size of the neuron (Eq. 3). Similarly, the concentration of proBDNF, 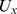, was:
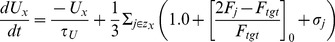
(7)where 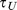 was the time constant of repulsive factor decay (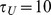 min).

#### Axon extension, branching and retraction

Growth and retraction of axons, and generation of synapses, was modulated by a segment's exposure to growth and trophic factors (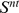, e.g., NGF, BDNF) and repulsive factors (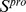, e.g., proBDNF):
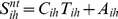
(8)

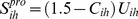
(9)where 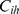 was the chemoaffinity of segment 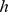 (Eq. 2) of neuron 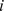, 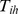 and 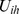 were the amount of constitutively released growth and repulsive factor in the vicinity of 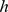 (Eqs. 6 and 7), and 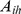 was the average trophic feedback received by synapses nearby to segment 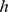. Specifically, 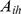 was the summation of 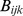 (Eq. 12) from all synapses on the segment plus the 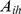 values of nearby segments, scaled by a distance factor 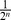 (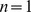 for the adjacent segment, 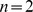 for a segment twice removed, etc).

The probability, 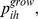 of axon segment 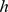 of neuron 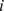 growing (or branching) was:
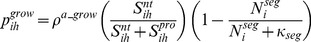
(10)where 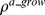 was the base probability for axon extension (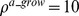 min^−1^) or branching (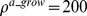 min^−1^), 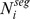 was the number of extant segments from neuron 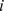 not including the initial axon extension into the colliculus (the initial trunk was omitted to help equalize growth in axons targeting anterior and posterior colliculus), and 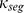 was a parameter regulating the size of the arbor (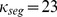, which was the number of axon segments required to stretch 25% the length of the colliculus). Segments were able to branch only after they grew (extended). Growth occurred along the trajectory of the axon, with minor variation, and branching was orthogonal to the axon's trajectory. To limit excessive rates of growth, each RGC could generate a maximum of 5 new segments every 10 minutes. This limitation had few qualitative effects on simulated retinocollicular development and is only present in the model to enable the model to more realistically simulate developmental paradigms when axons begin to branch when they first innervate the target structure (e.g., retinotectal; data not shown). To summarize the equation, the first bracketed term increases the growth probability with increased levels of growth factor and decreased levels of repulsive factors. The second bracketed term decreases axon growth probability with increasing axon size, as measured by the number of axon segments, helping to limit total growth. The form of both terms is intended to limit values to the interval [0,1] to be consistent with this being a probability.

Axon retraction occurred only at axon tips (i.e., only segments having no extensions or branches could retract). The probability of retraction, 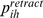, was:
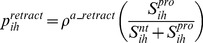
(11)where 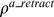 was the base probability for axon retraction (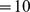 min^−1^).

#### Synapse growth and retraction

To implement synapses destabilization through usage, synapses were considered to release a destabilizing substance (e.g., proBDNF) on each presynaptic spike that, over the course of tens of milliseconds, destabilized the synapse. To stabilize synapses on the occurrence of a postsynaptic spike, the remaining unbound substance was converted into a growth-promoting substance (e.g., BDNF) which stabilized the synapse and promoted synapse and axon growth. This conceptual design was based on proBDNF being required for retinal axon ephrinA-mediated guidance [59]. Other implementations and conceptual descriptions to achieve this functional behavior are of course possible.

The effect of trophic factor on growth was represented by 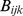, for synapse 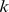 between neurons 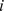 and 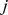. 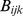 moved towards unity with higher levels of trophic feedback, and towards zero with less trophic feedback and more pro-neurotrophin exposure, and its value was updated on each pre- or postsynaptic spike:
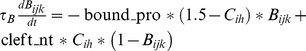
(12)where 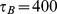 (spikes), 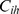 was the chemoaffinity of the axon segment (Eq. 2), 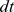 was the time since the previous pre- or postsynaptic spike, and for a postsynaptic spike:

bound_pro  =  cleft_pro *
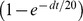


cleft_nt  =  cleft_pro - bound_pro
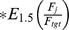


cleft_pro  = 0

while for a presynaptic spike:

bound_pro  =  cleft_pro *
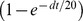


cleft_nt  = 0

cleft_pro  =  cleft_pro + 1 - bound_pro

The probability, 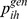, of an axon segment attempting to generate a synapse was:
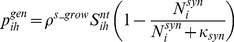
(13)where 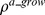 was the base probability of synapse generation (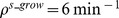), 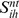 was the segment's exposure to trophic factor (Eq. 8), 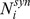 was the number of axonal synapses of neuron 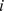, and 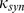 was a parameter regulating the size of the arbor (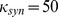). Each axon segment could have a maximum of four synapses and synapse generation in each axon segment was attempted every 500 ms.

When an axon segment created a synapse, a dendrite that spatially overlapped the segment was selected at random to receive the synapse. The target cell was then queried to determine if it would accept the synapse. The probability of acceptance was:
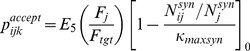
(14)where 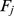 was the firing rate of postsynaptic cell 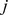, 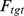 was the target firing rate of 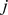, 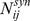 was the number of synapses between cells 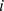 and 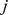, 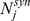 was the number of innervating synapses on 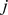, and 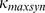 was the maximum ratio of innervating synapses from any given presynaptic neuron (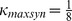). If the synapse was not accepted by the postsynaptic cell, it was destroyed.

Synapse survival was based on the trophic factor received by the synapse and that of nearby synapses. When considering synapse survival, the trophic support level, 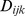, was:
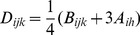
(15)where 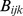 was the level of trophic factor received (Eq. 12), and 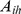 was a weighted average of 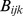 for all synapses near axon segment 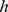 (see Eq. 8). In simulations where synapses were fully independent (Fig. 3), 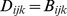, and in simulations where synapses were fully cooperative, 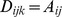.

When a synapse received less than an average amount of trophic feedback compared to other synapses on the axon, its probability of retraction was:
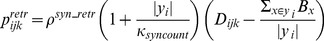
(16)where 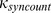 regulated the rate of retraction based on the number of existing synapses (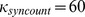 synapses), 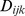 was the trophic support for the synapse (Eq. 15), 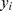 was the set of axonal synapses on 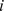.

Similarly, when a synapse received less than an average amount of trophic feedback compared to other synapses on the dendrite, its probability of retraction was:
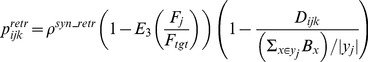
(17)where 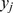 was the set of dendritic synapses on 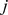. Synapses were subject to retraction from Eqs. 16 and 17 every 500 ms.
